# Expanding the Mitogen-Activated Protein Kinase (MAPK) Universe: An Update on MAP4Ks

**DOI:** 10.3389/fpls.2020.01220

**Published:** 2020-08-07

**Authors:** Lixia Pan, Ive De Smet

**Affiliations:** ^1^Department of Plant Biotechnology and Bioinformatics, Ghent University, Ghent, Belgium; ^2^VIB Center for Plant Systems Biology, Ghent, Belgium

**Keywords:** signaling, kinases, MAP4Ks, phosphorylation, development, stress

## Abstract

Phosphorylation-mediated signaling cascades control plant growth and development or the response to stress conditions. One of the best studied signaling cascades is the one regulated by MITOGEN-ACTIVATED PROTEIN KINASEs (MAPKs). However, MITOGEN-ACTIVATED PROTEIN KINASE KINASE KINASE KINASEs (MAP4Ks) are hardly explored. Here, we will give a comprehensive overview of what is known about plant MAP4Ks and highlight some outstanding questions associated with this largely uncharacterized class of kinases in plants.

## Introduction

To regulate their growth and development or respond to stress conditions, plants use—like many other organisms—phosphorylation-mediated signaling cascades. The central enzymes in these cascades are kinases, which—*via* reversible phosphorylation—mediate protein folding (conformation), protein function and the regulation of enzymatic activities, define substrate specificity, and influence protein localization, complex formation, and degradation (Stone and Walker, 1995). The *Arabidopsis* genome contains more than 1,000 kinases (Wang et al., 2003; Wang et al., 2007; Dissmeyer and Schnittger, 2011), including a largely uncharacterized class of mitogen-activated protein kinase kinase kinase kinases (MAP4Ks) (Champion et al., 2004). Here, we will give a comprehensive overview of what is known about plant MAP4Ks and highlight some outstanding questions.

## The Canonical MAPK Signaling Module

**Figure 1:**
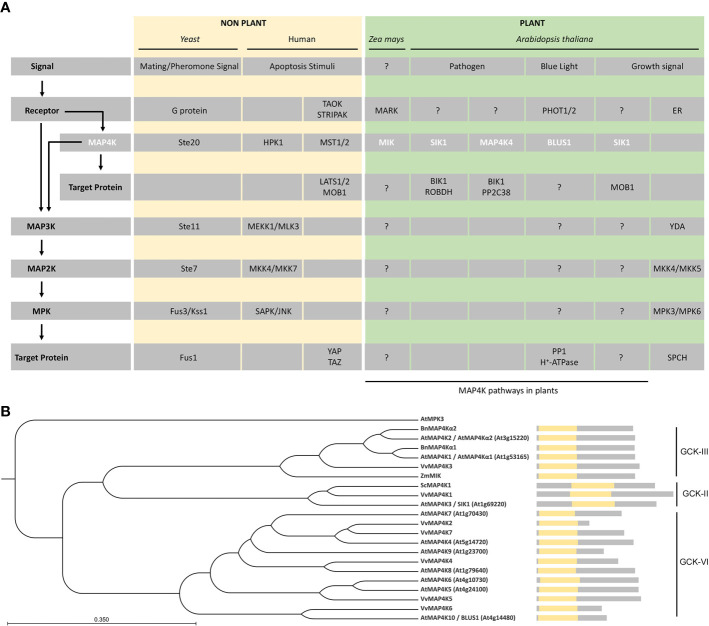
MAP4Ks in plants. **(A)** Representative examples of MAP4Ks in non-plants (pale gold) and plant (green) examples discussed in the text and their targets and signaling pathways. General protein classes, upstream signal, and possible regulatory pathways (arrows) are indicated on the left and relate to the specific protein names in the gray rows. Plant MAP4Ks are indicated in white.?, indicates missing knowledge (upstream regulation and/or downstream targets) in our understanding of indicated MAP4K pathways. **(B)** Phylogenetic tree of MAP4Ks in *Arabidopsis* and orthologs in other species discussed in the text. The tree was built using the UPGMA method and the Jukes–Cantor model (bootstrap analysis with 100 replicates) in the CLC Main Workbench 20.03 (CLC Bio-Qiagen, Aarhus, Denmark). A schematic of the protein sequence is shown, including the position of the kinase domain (yellow). The related subclass within the germinal center kinase (GCK) family is indicated.

One of the best studied signaling cascades is the one regulated by MITOGEN-ACTIVATED PROTEIN KINASEs (MAPKs). The MAPK cascade is conserved in yeast, insects, nematodes, plants, and mammals, and its main role is to modulate protein function through linear sequential serine/threonine and/or tyrosine phosphorylation (Leprince et al., 1999; Dan et al., 2001; Champion et al., 2004; Cakir and Kilickaya, 2015). The canonical MAPK signaling module is composed of a MAPK KINASE KINASE (MAP3K), a MAPK KINASE (MAP2K), and a MAPK (Dan et al., 2001; Colcombet and Hirt, 2008) (**Figure 1A**). In a typical MAPK cascade, a MAP3K specifically activates a dual specific MAP2K by phosphorylation, which in turn activates a MAPK by phosphorylation of threonine and tyrosine residues (Xu and Zhang, 2015; Krysan and Colcombet, 2018). Given the number of MAP3Ks, MAP2Ks, and MAPKs in *Arabidopsis thaliana* (Jonak, 2002; Champion et al., 2004; Colcombet and Hirt, 2008; Krysan and Colcombet, 2018), the theoretical MAPK network contains an enormous number of possible combinations.

Plant MAPK modules play important roles in regulating growth and development as well as responses to biotic and abiotic stresses (Mishra et al., 2006; Xu and Zhang, 2015). The sensors/receptors responsible for the activation of most plant MAPK modules are unknown (Xu and Zhang, 2015; Krysan and Colcombet, 2018). However, plant MAPK cascades are key modules downstream of receptor-like protein kinases (RLKs) or G-proteins (Cho et al., 2008; Cheng et al., 2015; Meng et al., 2015a; Liu and Zhou, 2018; Zhu et al., 2019). Related to developmental processes in *Arabidopsis*, HAESA (HAE) and HAESA-LIKE 2 (HSL2) function upstream of the MKK4/MKK5-MPK3/MPK6 module to regulate floral abscission and lateral root emergence (Cho et al., 2008; Zhu et al., 2019) and, for stomata development, ERECTA family members, SOMATIC EMBRYOGENESIS RECEPTOR KINASEs (SERKs) and TOO MANY MOUTHS (TMM) form a receptor complex that activates—upon ligand perception—the YDA-MKK4/MKK5-MPK3/MPK6 module, which leads to phosphorylation of the transcription factor SPEECHLESS (SPCH) (Lau and Bergmann, 2012; Meng et al., 2015b) (**Figure 1A**). With respect to biotic stress in *Arabidopsis*, FLAGELLIN-SENSITIVE 2 (FLS2) in a complex with BRASSINOSTEROID INSENSITIVE 1-ASSOCIATED KINASE 1 (BAK1) promotes the MAPKKK8–MKK1/2–MPK4 cascade and the receptor-like cytoplasmic kinase BRASSINOSTEROID-SIGNALING KINASE 1 (BSK1) directly associating with the FLS2–BAK1 complex, and the MAPKKK5–MKK4/MKK5–MPK3/MPK6 cascade controls the response to flg22 (Chinchilla et al., 2007; Sopena-Torres et al., 2018; Yan et al., 2018; Yu et al., 2018). In the context of abiotic stress in *Arabidopsis*, CALCIUM/CALMODULIN-REGULATED RECEPTOR-LIKE KINASE 1 (CRLK1) and CRLK2 are activated by both chilling and freezing temperatures, which leads to rapid MAPKKK8 activation and sequential activation of MKK1/2 and MPK4. The CRLK1/2–MAPKKK8–MKK1/2–MPK4 pathway positively regulates cold stress responses by inhibiting the activity of MPK3 and MPK6, which phosphorylate and promote the degradation of the transcription factor Inducer of CBF Expression 1 (ICE1) (Zhao et al., 2017; Liu and Zhou, 2018). Finally, RACK1 proteins, such as RACK1A, RACK1B, and RACK1C, function as MAPK scaffold proteins and link upstream G proteins to a downstream MAPKKK8–MKK4/MKK5–MPK3/MPK6 module in PrpL/ArgC protease-triggered immune signaling in *Arabidopsis* (Cheng et al., 2015; Meng et al., 2015a; Su et al., 2015).

## A New Hope: The MAP4K Family in Plants

However, in contrast to the activation mechanisms described above, a MAP3K is sometimes phosphorylated by a MAP4K (Dan et al., 2001; Champion et al., 2004) (**Figure 1A**). For example, yeast Ste20 acts as a MAP4K that directly phosphorylates Ste11, a MAP3K in the mating pathway (Wu et al., 1995), the mammalian Hematopoietic progenitor kinase (HPK1) phosphorylates the MAP3K MEKK1 (Dan et al., 2001; Chuang et al., 2016), and PAK2 activates the MAP3K Raf-1 by direct phosphorylation (Dan et al., 2001; Chuang et al., 2016). The yeast Ste20-related MAP4K family is evolutionarily conserved (Wu et al., 1995; Champion et al., 2004). In non-plant organisms, the MAP4K family is divided in two families according to the location of the kinase domain: (i) p21-activated kinases (PAKs), with a C-terminal kinase domain and an N-terminal GTPase-binding domain, and (ii) germinal center kinase (GCKs), with an N-terminal kinase domain and lacks the GTPase-binding domain (Dan et al., 2001).

However, to complicate matters, some MAP4Ks act as a MAP3K or phosphorylate proteins outside a MAPK module (**Figure 1A**). For example, in humans, the MAP4Ks Thousand-and-one Amino Acid 1 (TAO1) and TAO2 phosphorylate MKK3 and MKK3 or MKK6, respectively, to activate the p38 MAPK pathway (Dan et al., 2001; Chen et al., 2003), and in the mammalian Hippo signaling pathway, the MAP4Ks Mammalian STE20-like 1 (MST1) and MST2 phosphorylate and activate the Large Tumor Suppressor 1 (LATS1)/LATS2–MOB Kinase Activator 1A (MOB1A)/MOB1B complex to regulate cell proliferation, migration, and survival (Plouffe et al., 2016; Bae and Luo, 2018; Chen et al., 2019).

In plants, MAP4Ks were initially identified and described in *Brassica napus* (Leprince et al., 1999) and later also in *Zea mays*, *Solanum chacoense*, and *Vitis vinifera* (Llompart et al., 2003; Castells et al., 2006; Major et al., 2009; Cakir and Kilickaya, 2015).

In *Brassica napus*, *BnMAP4Kα1* and *BnMAP4Kα2* were isolated from a globular/heart stage embryo cDNA library (Leprince et al., 1999). These two putative proteins contain 12 serine/threonine protein kinase catalytic subdomains at the N-terminus, possess long disordered sequences at the C-terminus, and are similar to the GCK subfamily of yeast Ste20-like MAP4Ks (Leprince et al., 1999) (**Figure 1B**). *BnMAP4Kα1* and *BnMAP4Kα2* are mainly expressed in flower buds, siliques, different stages of embryogenesis, and roots (Leprince et al., 1999).

MAIZE ATYPICAL RECEPTOR KINASE (MARK)-INTERACTING KINASE (MIK), a GCK-III subfamily MAP4K from *Zea mays*, possesses an N-terminal kinase domain that displays high similarity to the GCK subfamily of yeast Ste20-like MAP4Ks (Llompart et al., 2003) (**Figure 1B**). Moreover, the kinase domain of MIK contains a VGTPFWMAPEV sequence, which aligns with the signature motif of Ste20-like kinases (Dan et al., 2001; Llompart et al., 2003), and both the N-terminal kinase domain and the C-terminal part share high sequence similarity with AtMAP4Kα1, AtMAP4Kα2, BnMAP4kα1 and BnMAP4kα2 (Llompart et al., 2003). MIK interacts with MARK, which is an atypical receptor kinase expressed during embryogenesis and in the meristems of adult maize plants, and this interaction increases the activity of MIK (Llompart et al., 2003; Castells et al., 2006) (**Figure 1A**). Different isoforms of MIK show variable kinase activity and are differentially activated through the interaction with MARK, suggesting that the kinase activity of MIK is also regulated by alternative splicing (Castells et al., 2006).

In *Solanum chacoense*, *ScMAP4K1* is expressed during fertilization and early embryogenesis (Major et al., 2009). Phylogenetic analysis revealed that ScMAP4K1 belongs to the GCK-II subfamily and is the ortholog of *Arabidopsis* SIK1/MAP4K3 (Major et al., 2009). However, unlike the GCK-II members in mammals, ScMAPK1 has a central kinase domain (Major et al., 2009). While *ScMAP4K1* is strongly expressed in reproductive tissues (such as pollen and pollen tubes) (Wu et al., 1995), full length *ScMAP4K1* is not present in both pollen and pollen tubes, suggesting that *ScMAP4K1* is also regulated by alternative splicing, similar to MIK in maize (Castells et al., 2006; Major et al., 2009). Interestingly, *ScMAP4K1* RNAi lines show altered ovule, seed and fruit development, indicating that ScMAP4K1 plays a vital role in those processes (Major et al., 2009).

## The *Arabidopsis* MAP4Ks Awaken

There are 10 MAP4Ks in *Arabidopsis* (Champion et al., 2004), but while plant MAP4Ks have been described already at the end of the 20^th^ century, it is only since 2013 that functional characterization in *A. thaliana* has begun (Takemiya et al., 2013a, Xiong et al., 2016). Most of the *Arabidopsis* MAP4Ks have an N-terminal catalytic kinase domain, but SERINE/THREONINE KINASE 1 (SIK1)/MAP4K3 has a more centrally located kinase domain (Xiong et al., 2016; Zhang et al., 2018) (**Figure 1B**). Phylogenetic analysis based on the MAP4K kinase domain showed that MAP4Kα1 and MAP4Kα2 are GCK-III subfamily members, that SIK1 belongs to the GCK-II subfamily, and that other *Arabidopsis* MAP4Ks are part of the GCK-VI subfamily (Major et al., 2009) (**Figure 1B**).

**Figure 2:**
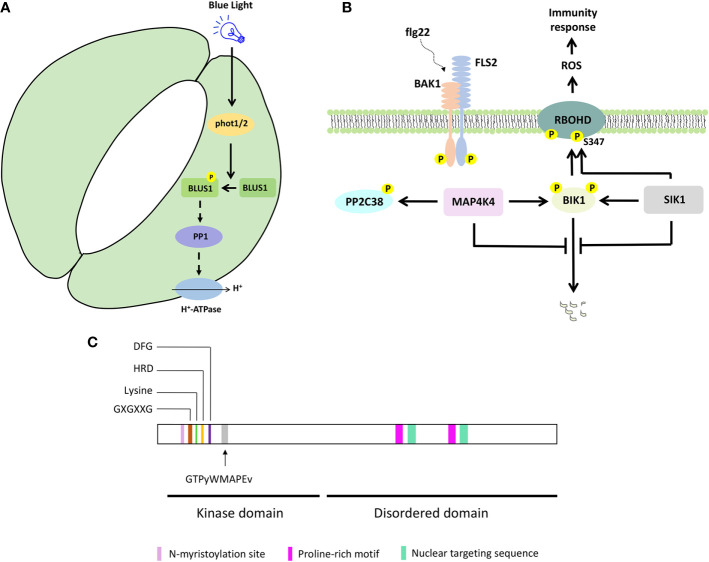
Selected MAP4K signaling pathways and protein features. **(A)** BLUS1/MAP4K10 signaling to control blue light-induced stomatal opening. **(B)** Simplified SIK1/MAP4K3 and MAP4K4 signaling to control immunity response. **(C)** MAP4K protein features on a hypothetical protein sequence. The kinase and disordered domain are indicated, together with the key features in the kinase domain and the consensus sequence associated with a MAP4K. Proline-rich motif, nuclear targeting sequence, and N-myristoylation site were shown for BnMAP4K1 and BnMAP4K2.

To control stomata opening, phototropins (PHOT1 and PHOT2) activate H^+^-ATPase, through PROTEIN PHOSPHATASE 1 (PP1) (Kinoshita and Shimazaki, 1999; Kinoshita et al., 2001; Takemiya et al., 2006, Takemiya et al., 2013b). Genetic and biochemical analyses revealed that PHOT1/2 phosphorylate BLUE LIGHT SIGNALING 1 (BLUS1)/MAP4K10 at the conserved Ser-348 in response to blue light, and this phosphorylation is important for BLUS1 function to regulate blue light-induced stomata opening (Schnabel et al., 2018; Takemiya et al., 2013a) (**Figures 1A** and **2A**). The *blus1* mutant stomata do not open in response to blue light but respond to an H^+^-ATPase activator. Furthermore, blue light-induced phosphorylation of BLUS1 at Ser-348 is absent in the *phot1 phot2* double mutant, and a BLUS1 phosphorylation dead (S348A) protein variant cannot complement the *blus1* mutant phenotype. Interestingly, an inhibitor of PP1, which mediates the signaling between PHOT1/2 and H^+^-ATPase, suppresses H^+^-ATPase phosphorylation but does not affect BLUS1 phosphorylation, suggesting that BLUS1 precedes PP1 in the signaling pathway leading to stomatal opening (Takemiya et al., 2013a; Takemiya and Shimazaki, 2016). However, there is likely also a BLUS1-independent pathway regulating H^+^-ATPase activity downstream of PHOT1/2 (Kostaki et al., 2020).

*Arabidopsis SERINE/THREONINE KINASE 1* (*SIK1*)/*MAP4K3* encodes a MAP4K kinase that contains a central kinase domain and that is highly conserved in land plants (**Figure 1B**) (Zhang et al., 2018). SIK1 complements the *ste20Δ* yeast mutant phenotypes with respect to bud site selection and mitotic exit (Xiong et al., 2016), confirming that SIK1 is similar to Ste20 and belongs to the MAP4K family (Xiong et al., 2016). *SIK1* is strongly expressed in mature organs and tissues, such as developed vascular tissues, stipules of true leaves, mature trichrome, and guard cells (Xiong et al., 2016). The *sik1* mutants are dwarfed and grow slow compared to wild type and display a shorter primary root and shorter root hairs, a reduced rosette leaf area, reduced area and number of the petal cells of fully opened flower, and reduced plant height, smaller siliques, and smaller seeds (Xiong et al., 2016). The above-mentioned *sik1* mutant phenotypes are caused by a reduced cell number and size, indicating that SIK1 influences growth *via* regulating cell proliferation and cell expansion (Xiong et al., 2016). In agreement with this, the expression of *SIK1* orthologs *BnMAP4Kα1* and *BnMAP4Kα2* is cell cycle-dependent and follows the same expression pattern as a G2-phase marker (Leprince et al., 1999). Thus, resembling the function of Ste20, SIK1 plays a role as a positive regulator of cell cycle exit (Xiong et al., 2016).

Interestingly, the N-terminal part of SIK1 interacts with MOB KINASE ACTIVATOR 1A (MOB1A) and MOB1B (Xiong et al., 2016). In mammals, Mob1 is involved in cell proliferation and tumor suppression and controls appropriate cell numbers and organ size (Pan, 2010). Also in Arabidopsis, *MOB1A* regulates organ growth, root tip tissue patterning, cell number and size, and sporophyte and gametophyte development (Galla et al., 2011; Pinosa et al., 2013). SIK1 is localized at the plasma membrane and in the trans-Golgi network/early endosome (TGN/EE), and the interaction of SIK1 and MOBs results in translocation of SIK1 to the nucleus (Xiong et al., 2016).

In humans, MAP4Ks are involved in immunity and activate nuclear factor kB immune signaling (Chuang et al., 2016). Indeed, also in Arabidopsis, MAP4Ks such as SIK1 and MAP4K4 function in immunity signaling (Zhang et al., 2018; Jiang et al., 2019) (**Figures 1A** and **2B**). Both SIK1 and MAP4K4 directly interact with, phosphorylate, and stabilize the immunity regulator BOTRYTIS-INDUCED KINASE 1 (BIK1) (Zhang et al., 2018; Jiang et al., 2019). Moreover, SIK1 directly interacts with and phosphorylates RBOHD upon flg22 perception (Zhang et al., 2018). In addition, PP2C38, a protein phosphatase, dephosphorylates BIK1 and maintains a minimum BIK1 phosphorylation status in the absence of flg22 (Couto et al., 2016). MAP4K4, which is localized in the cytosol and at the plasma membrane, phosphorylates PP2C38 in the presence of flg22, and phosphorylated PP2C38 disassociates from BIK1, indicating that MAP4K4 also regulates BIK1 activity through controlling PP2C38 (Jiang et al., 2019).

## The Rise of MAP4K Features

Plant MAP4Ks obviously contain several kinase-specific features, such as (i) an invariant lysine residue, an HRD motif, and a DFG motif, which contribute to ATP binding and regulate the catalytic activity of the protein kinase (Carrera et al., 1993; Jiang et al., 2019; Takemiya et al., 2013a; Xiong et al., 2016; Zhang et al., 2018), (ii) a glycine-rich loop with a GXGXXG motif, a structural hallmark of protein kinases (Zhang et al., 2018) (**Figure 2C**). In addition, the GTPyWMAPEv motif (a small letter indicates less conservation) in subdomain VIII of the kinase domain, termed the Ste20 signature sequence, is the primary reason to classify them as MAP4Ks (Dan et al., 2001; Jonak, 2002; Llompart et al., 2003) (**Figure 2C**). Furthermore, most plant MAP4Ks possess a long C-terminal region that is largely disordered (Champion et al., 2004; Major et al., 2009) (**Figure 2C**). Ste20 kinases also often contain proline-rich motifs (Leprince et al., 1999; Chuang et al., 2016; Miller et al., 2019), and these were also described for BnMAP4K1 and BnMAP4K2 (Leprince et al., 1999). While some non-plant MAP4Ks contain a C-terminal citron-homology domain (Chuang et al., 2016), this was not reported for plant MAP4Ks.

BnMAP4Kα1 and BnMAP4Kα2 also contain an N-myristoylation site (GxxxS/Txxx) between residues 12 and 19, which could imply membrane association, and two conserved SH3 binding domains at the C-terminus with proline-rich sequences that can bind SH3-containing adaptor proteins (Leprince et al., 1999) In addition, BnMAP4Kα1 possesses two nuclear targeting sequences (PQSRERR and RRGNARERLGNGKVNKR) (Leprince et al., 1999) (**Figure 2C**).

## Conclusion and Perspectives

While our knowledge on plant MAP4Ks is gradually increasing, there is still very little known about them (**Figure 1A**). One of the main outstanding questions—also for non-plant MAP4Ks—is what their substrates are and if they thus can all be considered as true MAP4Ks. More and more evidence is accumulating that these MAP4Ks also have other targets than MAP3Ks (Plouffe et al., 2016; Xiong et al., 2016; Bae and Luo, 2018; Zhang et al., 2018; Jiang et al., 2019). And, there is—so far—no evidence that plant MAP4Ks act on MAP3Ks. Taken together, this might warrant revisiting the MAP4K name. Second, we lack a comprehensive overview of the MAP4Ks in the green lineage, which would facilitate defining common characteristics. Third, using current methodologies to explore kinase signaling (Zhang et al., 2016) is an essential next step to deepen our insight not only regarding the substrates, but also with respect to potential protein complexes that are formed. Fourth, the subcellular localization of MAP4Ks spans the plasma membrane, cytoplasm, and nucleus, but the precise role in these compartments has hardly been investigated.

## Author Contributions

LP and IS organized and wrote the manuscript.

## Funding

LP was supported by a grant from the Chinese Scholarship Council.

## Conflict of Interest

The authors declare that the research was conducted in the absence of any commercial or financial relationships that could be construed as a potential conflict of interest.
